# A Study Protocol for Developing a Pragmatic Aetiology-Based Silicosis Prevention and Elimination Approach in Southern Africa

**DOI:** 10.3390/mps9010012

**Published:** 2026-01-14

**Authors:** Norman Nkuzi Khoza, Thokozani Patrick Mbonane, Phoka C. Rathebe, Masilu Daniel Masekameni

**Affiliations:** 1 Department of Environmental Health, Faculty of Health Sciences, University of Johannesburg, Johannesburg, South Africa; tmbonane@uj.ac.za (T.P.M.); prathebe@uj.ac.za (P.C.R.); 2 Human Capital and Institutional Development, African Union Development Agency-NEPAD, Johannesburg, South Africa; 3 Department of Development Studies, University of South Africa (UNISA), Pretoria, South Africa; danielmasekameni@gmail.com

**Keywords:** silica dust, Fourier transform infrared spectroscopy, artisanal small-scale mines, Multiple-Path Particle Dosimetry Modelling

## Abstract

Workers’ exposure to silica dust is a global occupational and public health concern and is particularly prevalent in Southern Africa, mainly because of inadequate dust control measures. It is worsened by the high prevalence of HIV/AIDS, which exacerbates tuberculosis and other occupational lung diseases. The prevalence of silicosis in the region ranges from 9 to 51%; however, silica dust exposure levels and controls, especially in the informal mining sector, particularly in artisanal small-scale mines (ASMs), leave much to be desired. This is important because silicosis is incurable and can only be eliminated by preventing worker exposure. Additionally, several studies have indicated inadequate occupational health and safety policies, weak inspection systems, inadequate monitoring and control technologies, and inadequate occupational health and hygiene skills. Furthermore, there is a near-absence of silica dust analysis laboratories in southern Africa, except in South Africa. This protocol aims to systematically evaluate the effectiveness of respirable dust and respirable crystalline silica dust exposure evaluation and control methodology for the mining industry. The study will entail testing the effectiveness of current dust control measures for controlling microscale particles using various exposure dose metrics, such as mass, number, and lung surface area concentrations. This will be achieved using a portable Fourier transform infrared spectroscope (FTIR) (Nanozen Industries Inc., Burnaby, BC, Canada), the Nanozen DustCount, which measures both the mass and particle size distribution. The surface area concentration will be analysed by inputting the particle size distribution (PSD) results into the Multiple-Path Particle Dosimetry Model (MPPD) to estimate the retained and cleared doses. The MPPD will help us understand the sub-micron dust deposition and the reduction rate using the controls. To the best of our knowledge, the proposed approach has never been used elsewhere or in our settings. The proposed approach will reduce dependence on highly skilled individuals, reduce the turnaround sampling and analysis time, and provide a reference for regional harmonised occupational exposure limit (OEL) guidelines as a guiding document on how to meet occupational health, safety and environment (OHSE) requirements in ASM settings. Therefore, the outcome of this study will influence policy reforms and protect hundreds of thousands of employees currently working without any form of exposure prevention or protection.

## 1. Introduction

Crystalline silica exposure is a well-known health risk in mining [1,2,3]. Dust control measures have significantly improved over the years in developed countries in Europe and North America, but the same cannot be said about developing countries [2]. The issue of workers’ exposure to crystalline silica in South African mining has received much-needed attention from the local and international media. This was particularly evident during the recent epidemic of silicosis coupled with tuberculosis in former miners that led to legal actions against mining companies [4]. Miners’ exposure to crystalline silica dust is well-documented in South Africa, contrary to other Southern African countries. About 9% of workers monitored in 2006 were exposed to silica dust at levels above the Department of Mineral Resources and Energy (DMRE) OEL of 0.1 mg/m^3^. Gold mines had the highest exposure, with an average exposure of 0.14 mg/m^3^; stope activities had an average exposure of 0.16 mg/m^3^; and the stope team leaders saw an average exposure of 0.27 mg/m^3^ [5]. The mean area respirable dust level was 1.2 mg/m^3^ in a study carried out in a stone-crushing environment in Tanzania [6]. In another study undertaken in Zambian quarries, the mean respirable dust level was 0.90 mg/m^3^ in two quarries [7]. The above-mentioned studies have reported a prevalence of inadequate dust control practices. There is a paucity of studies regarding respirable crystalline silica exposures and controls in the Southern African Development Countries (SADC). However, studies in Iran and India have reported mean exposure levels exceeding local and international standards by 1.24 mg/m^3^ and 39.7 mg/m^3^, respectively. Excavating and ripping and plant operators had a median respirable dust exposure of 1.0 mg/m^3^ in South African stone quarries [8]. Occupational respirable silica dust exposure remains a significant risk factor for pulmonary tuberculosis (PTB) in South Africa. Therefore, it is highly recommended that any PTB patients of working age be screened for silica exposure [9]. This will allow the early identification and institutionalisation of control measures.

Aetiology (or etiology) is the science of studying the causes of diseases or underlying environmental, biological, or occupational factors influencing a disease phenomenon; all diseases must have a cause to manifest, and so must silicosis [10]. A cause is a phenomenon capable of producing an effect. In environmental epidemiology, several types of causation are used to explain the development of health conditions, including necessary, sufficient, proximal, and distal causes. A necessary cause is a factor that must be present for a specific health condition to arise. A sufficient cause, on the other hand, refers to a combination of factors or events that, when occurring together, are enough to produce a particular disease, injury, or adverse health effect. A proximal cause is an immediate and direct precipitating factor, and a distal cause is a distant or remote cause of the phenomenon. These are concepts in epidemiology [11], which is defined as the study and analysis of disease distribution and determinants/risk factors in a population. Epidemiology and its concepts shape evidence-based policies and practices by thoroughly identifying disease risk factors to design effective preventive interventions. Therefore, understanding these concepts aids us in developing effective and efficient intervention strategies.

Building on the understanding of necessary and sufficient causes, it is also important to consider multifactorial causation in environmental epidemiology. This concept refers to the idea that multiple, and often varying, combinations of factors may be required to bring about a specific health outcome [12]. Among these are predisposing factors, which do not directly cause a disease but rather increase an individual’s susceptibility. These factors include genetic makeup, age, sex, and existing health conditions, and they shape how an individual might react to environmental agents, personal interactions, or stimuli. Although such factors are often necessary for disease occurrence, they are rarely sufficient on their own. These factors may be “necessary” but are rarely “sufficient” to cause the disease. In the analysis of health-related phenomena, precipitating factors refer to immediate elements or events that trigger the onset of a disease, illness, injury, behavioural reaction, or course of action. These factors often serve as the final catalyst that sets a pathological process into motion. When multiple precipitating factors are present, one typically stands out as more critical or more clearly identifiable than the others and may even be considered a “necessary” condition for the phenomenon to occur. For instance, in a person with multiple risk factors for cardiovascular disease, an acute stress event or physical exertion might act as the precipitating trigger for a heart attack. Risk factors refer to individual behaviours, lifestyle choices, environmental exposures, or genetic traits that are statistically linked to an increased likelihood of developing specific health conditions that are considered significant for prevention efforts. These factors do not necessarily cause disease directly, but their presence elevates the probability of adverse health outcomes [11]. It is imperative to grasp the above concepts in order to design and implement efficient and effective control of toxic dust elements in any environment, including occupational settings with varying environmental conditions.

There are four principles for particle deposition mechanisms in the lungs, that is, four means by which particles of different sizes are deposited in human lungs: interception, impaction, sedimentation, and diffusion. Interception occurs when particles are intercepted and deposited as they travel close to the surface of the airway passages. It is important to note that fibre length determines the area of deposition, e.g., fibres between 1 µm and 200 µm in length are deposited in the bronchial tree. Impaction occurs when suspended airborne particles tend to travel along their path and stick to the airway systems when there is a bend/curve, as it maintains their original air movement path. Air velocity and particle mass determine the likelihood of impact. Any particle greater than 10 µm (aerodynamic diameter) cannot penetrate the lower tissues of the respiratory tract; as a result, they are deposited in the nose or throat instead. This applies to spherical particles with similar settling velocities regardless of shape, size, or density, and it is imperative to compare how they will settle out in the airflow stream.

**Figure 1:**
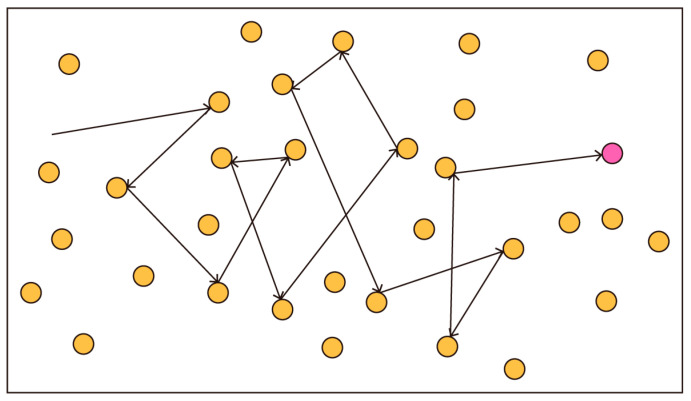
Brownian motion, reproduced from [16] with permission from Nagwa, 2025.

Sedimentation occurs when airborne particles lose their buoyancy (their ability to stay in the air) due to gravitational force and air resistance. The particles settle or are deposited mainly in the bronchi and the bronchiole section of the lung. It is important to note that sedimentation is negligible when the particle’s aerodynamic diameter is less than 0.5 µm. In diffusion, when particles smaller than 0.5 µm are in random motion, they are deposited on the lung walls by chance. This movement is also known as “Brownian motion”. The smaller the particle size, the more intense their movement. This is the most critical mechanism of deposition in the small airways and alveoli, as submicron particles of a size equal to or less than 001 µm can also be trapped in the upper respiratory tract too, due to random motion [13,14]. Therefore, decreasing particle size means reducing sedimentation efficiency, for example, while increasing the intensity of Brownian diffusion (Figure 1). The deposition criterion for a spherical particle is a function of the position of the centre of mass only [15]. The effective control of workers’ exposure to respirable crystalline silica dust requires a basket of controls rather than a single approach or a singular hierarchy of controls. Engineering controls remain the key targeted control methodology to reduce specific and sometimes difficult or small dust particles that are generally not easy to reduce or control due to varied properties and behaviours. Silicosis and silica dust health literacy is also important in interventions (for workers and inspectors).

This study will apply the principle of Occam’s razor: when competing hypotheses or explanations exist for a phenomenon, the one that makes the fewest assumptions or requires the least number of entities should be preferred. In other words, the most straightforward intervention leading to the desired results is usually the best. However, Occam’s razor is not a rule or law but a heuristic or guiding principle that helps evaluate the plausibility and validity of different arguments or interventions [17]. Consequently, the scientific literature informs us that submicron dust is toxic, and we argue that the most effective and efficient submicron dust monitoring and control technology is well understood by the people implementing it. This study aims to systematically evaluate the effectiveness of respirable dust and respirable crystalline silica dust exposure evaluation and control in the mining industry. The study objectives are as follows:

- To conduct a systematic literature review following the PRISMA framework of efficient dust control techniques for relevant exposure scenarios;
- To conduct a systematic desktop review of Southern African occupational health and safety laws, policies, and guidelines for the effective elimination of occupational lung diseases (silicosis);
- To determine baseline measurements of the workers’ exposure to respirable dust and respirable crystalline silica dust in the small-scale mining industry and analyse the current control measures;
- To develop harmonised regional hazardous substance occupational exposure limits (OELs) considering the available regional OELs and international best practices, in relation to the SADC protocol on mining and the objectives of the SADC employment and labour protocol;
- To assess the knowledge, attitudes, and practices of workers exposed to crystalline silica dust in the mines;
- To develop a cost-effective respirable silica dust management strategy based on the research findings.

### 1.1. Research Questions

- Is there an effective respirable crystalline silica dust management strategy within the SADC mining sector?
- Does a harmonised regional hazardous substance occupational exposure limit system exist in the SADC?

### 1.2. Study Hypothesis

The lack of a formalised regional system for tracking occupational hazard exposure limits within the Southern African Development Community (SADC) has resulted in divergent regulatory frameworks, which increase the danger to employee health and safety throughout the region. There is a notable correlation between the adoption of effective respirable crystalline silica dust control measures and reduced respiratory ailments among employees in the Southern African Development Community (SADC) mining industry. This study proposes that mining facilities equipped with robust ventilation systems, active silica monitoring, and workforce education programs will have lower healthcare costs associated with silica exposure, thereby supporting these management strategies across the sector.

## 2. Materials and Methods

### 2.1. Study Design

The researchers will conduct a field experimental, quantitative cross-sectional study to achieve the aims and objectives of the research (Appendix A).

### 2.2. Study Setting and Population

**Figure 2:**
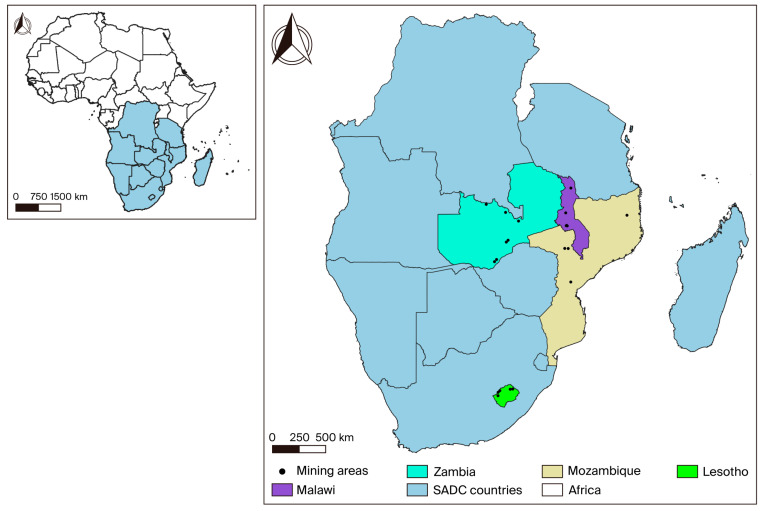
Presentation of the study sites (SADC countries).

Countries with a history of silicosis, tuberculosis, and other occupational lung diseases will be selected, as well as those with a long-standing mining tradition and those beginning mining activities. The study will be conducted in collaboration with the relevant ministries responsible for mines, labour, and health. The focus will be on four Southern African countries with significant interest in medium- to small-scale and artisanal mining: Lesotho, Malawi, Mozambique, and Zambia. Mines in districts implementing the Southern Africa Tuberculosis and Health System Support (SATBHSS) project were chosen for the study, as shown in Figure 2. These mines will be categorised into large-, medium-, and small-scale, including artisanal small-scale mines, and further subdivided by commodity. Sampling sites will be selected to ensure the representation of each major commodity. Lesotho has a total of 11 active mines; therefore, the sample will include two large mines (diamonds), one medium mine (sandstone), and two small mines (quarries), representing the primary commodities mined in the country. In Malawi, there is one large coal mine, two medium-sized limestone mines, and two artisanal and informal small-scale gemstone mines. Mozambique will contribute two large mines (coal and ruby), one medium mine (gold), and one small mine (quarry). In Zambia, there are three large mines (copper and coal), two medium mines (limestone and coal), and two small mines (quarries).

Samples will be collected from a total of 22 mines across these four countries. The study populations from the four countries will consist of mine workers employed in the following mining areas: diamonds, sandstone, and quarries. Participants will include males and females aged 18 years or older who are likely to be exposed to crystalline silica dust in environments where this exposure poses a significant occupational health risk. The study aims to encompass a diverse population, as these workers represent the workforce most likely to encounter silica dust during their daily mining activities. Given the nature of these mining operations, workers may experience varying levels of silica dust exposure depending on factors such as job roles, duration of exposure, ventilation systems, and adherence to occupational health and safety regulations. By including a broad demographic from multiple mining sectors, the study seeks to assess the extent of exposure, evaluate associated health risks, and provide insights into preventive measures necessary to mitigate the impact of silica dust inhalation on respiratory health and overall well-being.

### 2.3. Sampling and Sample Size

Stratified random sampling will be adopted; each country will form a stratum, and mines will be randomly selected. However, study participants will be conveniently selected from the mines until the sample size is reached. The researchers will approach the available miners on the day of data collection for recruitment purposes to participate in the study.

**Figure 3:**
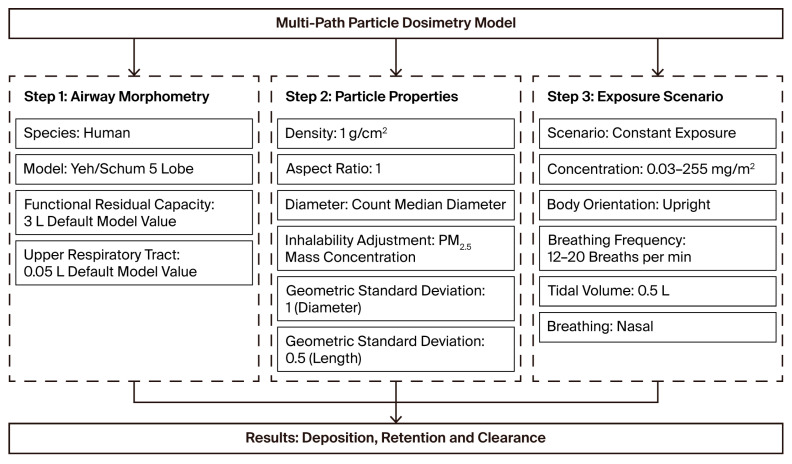
MPPD input parameters for lung deposition dose, retention, and clearance [18].

Respirable dust samples will be collected from mining sites in Lesotho, Malawi, Mozambique, and Zambia. The quantity of dust samples will be determined based on commodities, mine size, and job title/activity. Data collection will be conducted over a three-month period during the winter season. Two samples will be collected on two consecutive days at each site per participant, with the distribution of samples as follows: 80 samples from Lesotho, 90 from Malawi, 70 from Mozambique, and 140 from Zambia. In total, an estimated 380 samples will be collected, including 190 pre-intervention respirable crystalline silica dust samples and 190 post-intervention mass concentration samples, which will include particle size distribution samples obtained using a real-time monitoring system. The aerosol surface area, lung deposition concentrations, and doses will be estimated utilising the Multiple-Path Particle Dosimetry Model (MPPD) to assess aerosol deposition within the lungs (see Figure 3).

### 2.4. Data Collection

This study will rely on primary data collected from five Southern African countries, with a particular focus on medium- to small-scale mining operations, as well as artisanal small-scale mines (ASMs). These mining sectors are of significant interest due to their often-informal nature, limited regulatory oversight, and increased potential for hazardous exposures, particularly crystalline silica dust. Data collection will take place in Lesotho, Malawi, Mozambique, and Zambia, where mining plays a crucial role in economic development and employment. The study will encompass a diverse range of mining activities, including gemstone extraction, quarrying, and other mineral processing operations, with an emphasis on understanding workplace conditions, exposure levels, and health risks associated with silica dust inhalation. By targeting these countries, the study aims to generate region-specific insights that can inform policies, workplace interventions, and occupational health and safety strategies tailored to medium- and small-scale mining operations across the region.

#### Environmental Measurement

Respirable dust concentrations will be measured using a gravimetric sampling method to ensure the accurate quantification of airborne particulate matter. This method will employ a GillAir personal sampling pump, a device widely used in occupational hygiene studies for monitoring worker exposure to airborne contaminants. A Higgins–Dewell cyclone will be attached to the sampling system to effectively capture respirable dust fractions. The cyclone will be pre-calibrated to a flow rate of 2.2 L per minute (L/min), which is the optimal rate for selectively isolating respirable particles while preventing the collection of larger, non-respirable particles. Post-calibration checks will be conducted to verify and maintain accuracy throughout the sampling process. This approach ensures that only fine, respirable dust particles capable of penetrating deep into the lungs are collected for further analysis. The use of gravimetric sampling in conjunction with cyclone separation enhances the reliability of exposure assessment, providing crucial data for evaluating occupational health risks associated with airborne dust in mining environments. Measurements will be taken according to the Health and Safety Executive laboratory method for determining hazardous substances: “MDHS 14/3: General methods for sampling and gravimetric analysis of respirable and inhalable dust” [19]. The analysis of respirable quartz concentration will be conducted using “MDHS 101: Crystalline silica in respirable airborne dust: Direct-on-filter analysis by infrared spectroscopy and X-ray diffraction” [20]. The study will be carried out through a SANAS-accredited laboratory using both methods (MDHS 14/3 and MDHS 101).

Sample collection will be performed by the project investigator, who is a SAIOH-certified occupational hygiene technologist. The post-intervention samples will be analysed using an accredited laboratory and a portable FTIR analysis machine. Particle size distribution will be determined using Nanozen, providing particle size distributions (PSDs) in the range from submicrons to microns at sufficiently high resolution to achieve accurate data on small particle sizes. The alveolar LDSA concentration/dose will be estimated using the Multiple-Path Particle Dosimetry Model (MPPD) by entering the concentration data used to estimate the concentration of aerosol deposition in various areas of the lungs, as in Figure 3. This method will help determine the effectiveness of current controls in managing relevant toxic submicron dust.

The respirable dust samples will be analysed using a gravimetric analysis method according to HSE MDHS 14/4, and the analysis of RCS content on the filter will be quantified by X-ray diffraction as per HSE MDHS 101. All laboratory analyses will be conducted by a SANAS-accredited laboratory that conforms to ISO/IEC 17025 [21]. Values falling below the detection limit will be imputed using the established detection threshold, whereby values reported as below detection limit (BDL) or less than 0.01 mg will be replaced with 0.01 mg. This substitution method is consistent with recommended practices and has been shown to perform comparably to more complex techniques like maximum likelihood estimation. It offers a meaningful improvement over basic substitution or average-based methods. The process involves modelling the detected values using a log-normal distribution and generating predicted values for non-detects based on their position or rank relative to the fitted distribution. As a result, each undetected measurement is replaced with a value that reflects the underlying data distribution. Each non-detect, therefore, ultimately becomes replaced by a prediction. While the predictions do not have any individual meaning, the modified dataset can be processed in traditional software as if there were no non-detects.

### 2.5. Data Analysis

The collected data will be entered into Microsoft Excel^®^ spreadsheets for data cleaning, organisation, visualisation, and preliminary statistical computations. Thereafter, they will be entered into STATA Now/MP 19.5 for descriptive and inferential analysis. The row-wise deletion approach will be used to handle missing values in the dataset, which will avoid reducing variability and potentially introducing bias.

To comprehensively assess exposure levels, the study results will be compared against multiple occupational exposure limits (OELs) to evaluate compliance and potential health risks. These include the South African Occupational Exposure Limit (OEL1), which provides national regulatory guidelines for workplace exposure; the National Institute for Occupational Safety and Health (NIOSH) Permissible Exposure Limit (PEL) (OEL2), which establishes enforceable exposure standards in occupational environments; and the American Conference of Governmental Industrial Hygienists (ACGIH) Recommended Exposure Limit (REL) (OEL3), which offers scientifically based recommendations to protect worker health. A statistical hypothesis testing approach will be employed to evaluate the significance of differences in respirable crystalline silica (RCS) exposure levels across various occupations and industries. Exposure data will be assessed using multiple metrics, including Lung-Deposited Surface Area (LDSA), to provide a more nuanced understanding of potential health risks. Descriptive data will be presented in frequencies and percentages through tables and figures for nominal variables. Continuous variables will be reported as mean (with standard deviation), median, and range. A detailed analysis plan for objectives 3 and 5 is discussed below:

- To determine baseline measurements of the workers’ exposure to respirable dust and respirable crystalline silica dust in the small-scale mining industry and analyse the current control measures.

Assuming that the exposure data follow a log-normal distribution, the dataset will undergo a log-transformation, and appropriate parametric tests will be applied. To compare two groups, a t-test will be applied, while comparisons involving more than two groups will be assessed using Analysis of Variance (ANOVA). In instances where the data violate the assumption of normality, non-parametric alternatives will be used, specifically, the Mann–Whitney U test for two-group comparisons and the Kruskal–Wallis test for analyses involving multiple groups. The Wilcoxon Signed-Rank test will be used to evaluate pre- and post-intervention paired samples, aiming to identify statistically significant changes in exposure concentrations resulting from the implemented intervention measures.

- To assess the knowledge, attitudes, and practices of workers exposed to crystalline silica dust in the mines.

A linear regression model will be employed to determine factors influencing practice scores. A bivariate analysis will be performed to examine the relationship between the dependent variable (practice score) and the independent variables, including socio-demographic characteristics, knowledge, and attitudes. Independent variables showing a statistically significant association with the practice score will be incorporated into the subsequent multivariate analysis. Variables demonstrating significant associations in the final model will be considered influential regarding practices within the study population. The level of statistical significance will be set at 0.05.

## 3. Discussion and Expected Results

**Figure 4:**
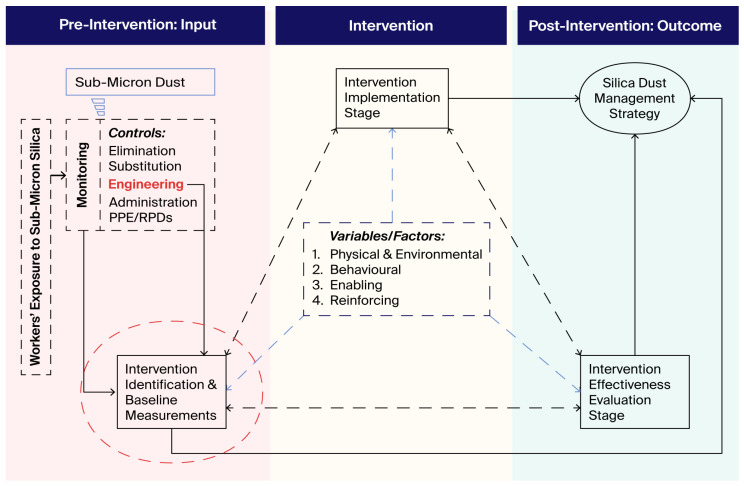
Conceptual framework for workplace exposure reduction intervention [16,23].

The main aim of any respirable crystalline silica dust control program is to eliminate the development of silicosis [22] and any other disease associated with exposure to respirable crystalline silica. Although silicosis is an incurable disease, it is preventable. The main strategy to curb the scourge of silicosis is to prevent workers’ exposure to RCS dust. The conceptual framework proposed in this study integrates three phases (intervention, implementation, and effectiveness evaluation) of the intervention research model developed by Goldenhar [23] as part of the National Occupational Research Agenda (NORA) of NIOSH. We developed a conceptual framework demonstrating the three stages of the study (see Figure 4). The research aims to identify and test assessment and dust suppression technologies to reduce worker exposure. The study will comprise five central tasks: gathering background information, developing partnerships, selecting methods and designs, implementing research and disseminating findings [23]. A few variables could influence the study’s reliability, i.e., physical and environmental factors, behavioural factors, and enabling and reinforcing factors that will be recognised during the study (see Figure 4).

The management strategy will be an outcome of the three phases of the research process. The South African mining sector continues to struggle to reduce occupational lung diseases; the country reported 1197 cases of occupational diseases, including tuberculosis (TB), in 2021, 1403 in 2022, 1592 in 2023, and 1456 in 2024. The government recorded a 13% and 19% increase in occupational diseases, including TB, in 2023 and 2024, respectively, against a 10% reduction target [24,25]. Silica dust measurement and control projects have been funded over the past few decades by SIMRAC, but silica exposure levels, coupled with silicosis and tuberculosis, remain a priority in mining [26]. In 2015, there were 835 diagnosed cases of silicosis [26]. The report continued to state that the 10-year average silica dust exposure measurement result, which was below the OEL, was 88.3% short of the MHSC target of 95%. This is due to the current method’s inefficiency in monitoring and controlling all dust particles, as well as the surface reactivity that determines the toxicity of crystalline silica dust [27].

**Figure 5:**
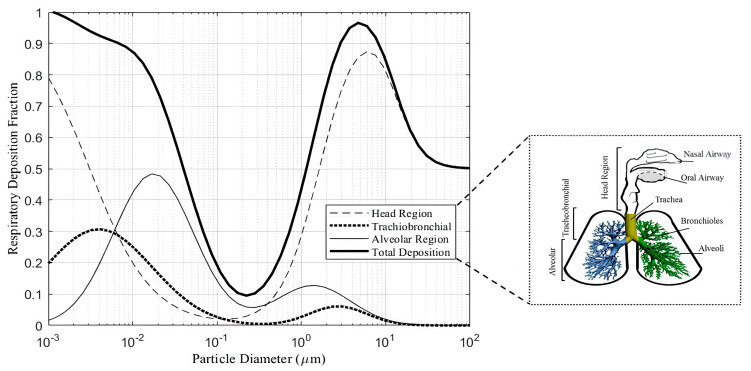
Probability of deposition of dust in the three regions of the lungs depends on the size of the particle [13].

The rate at which dust particles are deposited in the different areas of the respiratory system depends on the particle size of the dust. The human respiratory system is composed of the head region, the tracheobronchial (TB) region, and the alveolar region, also known as the gaseous exchange region (Figure 5) [13].

**Figure 6:**
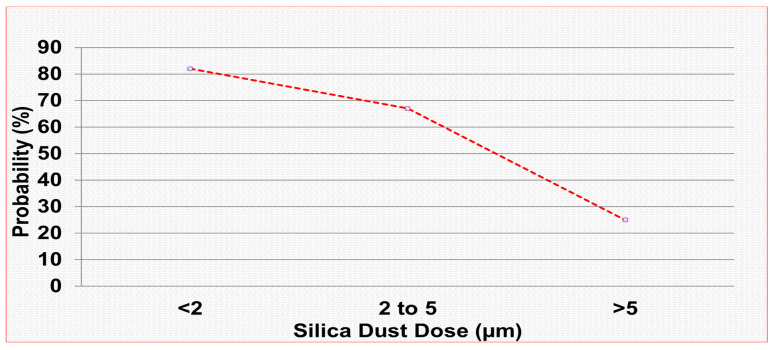
Respirable silica dust particle sizes and probability of silicosis in rats.

The surface area concentration or particle number concentration of dust retained in the alveoli would be an indicator of the severity of crystalline silica dust, as smaller particles occupy a relatively larger surface area of the lungs. In vivo studies have shown that quartz particle sizes of less than 2 µm have an 82% risk of causing silicosis in rats, 2–5 µm have a 67% risk, and greater than 5 µm have a 25% risk (Figure 6) [27,28].

Therefore, it is hypothesised that the surface area concentration or particle number concentration of the dust retained in the alveoli will indicate the severity of crystalline silica dust exposure, as smaller particles will occupy a larger surface area of the lungs compared to larger particles [27]. Therefore, the effective and sustainable management of submicron crystalline silica dust will reduce the incidence of occupational lung diseases in the region. Almost 86% of the African working population works in the informal sector [29]. ASMs represent a growing sector that employs more than 40 million people in Africa [30]. The prevalence of silicosis in small-scale mining ranges from 9% to 51% in Africa [31,32,33,34,35]. Approximately 49.5 million small-scale miners worldwide are exposed to high silica concentrations; it is, therefore, concerning that a large and growing population of ASM workers, along with their families and communities, are affected by high silica dust exposure [36]. Workers’ exposure to respirable silica dust increases the risk of tuberculosis, even without silicosis [37]. The Southern African region has a high burden of HIV infection and tuberculosis, requiring a comprehensive implementation of the hierarchy of controls. However, there are considerable gaps in the hierarchy of controls, as many countries lack policies that enable compliance, and many small-scale miners lack adequate resources to implement engineering controls effectively.

The Global Burden of Diseases (GBD) estimates that silicosis causes 32% of global pneumoconiosis [38]. Africa has recorded an increase in cases of pneumoconiosis, and African Union (AU) member states, such as Egypt, South Africa, Ethiopia, the Democratic Republic of the Congo (DRC), and Algeria, are the most affected countries [38,39]. Africa accounts for approximately 5% of the global pneumoconiosis mortality, with 20% attributed to silicosis. Southern Africa has the highest pneumoconiosis mortality rate and disability-adjusted life year (DALY) rate at 1.3/100,000 and 28.9/100,000, respectively [38,39]. Although studies in South Africa indicate a decline in the mining labour force, more mine workers are migrating to mines outside South Africa, where occupational health and safety laws and standards are often limited or poorly enforced, with inadequate enforcement capacity [40]. These results, coupled with an increase in informal and artisanal small-scale mining operations, increased mining activities, complexity of mining methodological requirements, and introduction of new mining technologies and new diseases all call for a regional harmonisation of standards, multisectoral approach and stakeholder proactiveness in identifying and using innovative monitoring and control mechanisms for toxic respirable silica dust, including targeted prevention methodologies [39,41].

The findings from the proposed study will be communicated to regulatory authorities, participating mines, and the miners involved in the study. Furthermore, the findings will be published and presented at conferences and in peer-reviewed journals. Moreover, a thesis will be available on the University of Johannesburg website (see Table 1).

## 4. Conclusions

Airborne dust prevention stops dust from becoming airborne, and airborne dust suppression captures airborne dust released into the atmosphere, causing the particles to collide, agglomerate, and fall [42]. Studies have been conducted on silica dust measurements and controls. Dust control technologies have been introduced to reduce workers’ exposure to silica dust. However, Kissell noted that most dust control methods for underground environments achieve only a 25% to 50% reduction in respirable-sized dust, which is insufficient for compliance [43,44,45], let alone for preventing disease development. South African mining groups have identified and adopted various dust suppression systems that control dust through wetting and spraying. However, very little effort has been made to investigate the efficacy of such controls against submicron particles. Moreover, the workplace exposure assessment criteria require gravimetric dust analysis techniques, which are expensive and time-consuming, and necessitate the services of highly qualified occupational or industrial hygienists. In contrast, the real-time worker exposure monitoring technique is the opposite of the gravimetric method, underscoring the need for and importance of this study.

Internal deposition following the inhalation of airborne silica dust will be modelled using the Multiple-Path Particle Dosimetry (MPPD) model. We will determine whether the silica dust particles are small enough to be deposited in the respiratory system. Additionally, we will determine the effectiveness of controls in reducing the deposition fractions of the inhaled particles in the head, thoracic, and gas-exchange regions of the human respiratory system [45].

The envisaged limitations include acceptance of the proposed methods by regulatory authorities, mines, and mine workers, as well as access to mines. Additionally, environmental factors such as rainy and windy conditions can affect sampling in all operations, more so at ASMs and open-cast mines. The sustainability of the proposed methodology, particularly in artisanal small-scale mines, should also be examined further.

## Figures and Tables

**Table 1:** Research communication plan.

Delivery Description	Purpose	Target Audience	Delivery Method	Frequency/Number	Communication Content	Responsible
Information/awareness sessions	Mandatory information session	Mines, workers, research assistants, etc.	Meetings and one-on-one sessions.	Continuous	Study the consent, benefits, and drawbacks.	Principal investigator
Status reports	Mandatory in case of high exposures	Regulatory authorities, participants, etc.	Presentations, status brief reports, workers, and management.	Monthly for 4 months	Progress, challenges, overexposures, and pre-intervention.	Principal investigator
Local research outcome session	Information session to raise awareness	Chamber of Mines, professional organisations, workers, etc.	Toolbox and induction sessions.	Four sessions, one session per country	Results, recommendations, monitoring, selection and use of respiratory protective devices, symptoms, and health effects of exposure.	Principal investigator
Regional and continental	Policy forums, information sessions	SADC and AU steering committees, etc.	Policy briefs, presentations, session reports, etc.	Two regional and continental sessions	Policy briefs and presentation of the study results, and control measures, including action plans.	Principal investigator
International	Mandatory as required by the study protocol	International conferences, peer-reviewed journals, etc.	Conferences, peer-reviewed papers, etc.	Target four international presentations	Conference presentation and proceedings, peer-reviewed, published, and draft papers.	Principal investigator and supervisors

**Figure A1:**
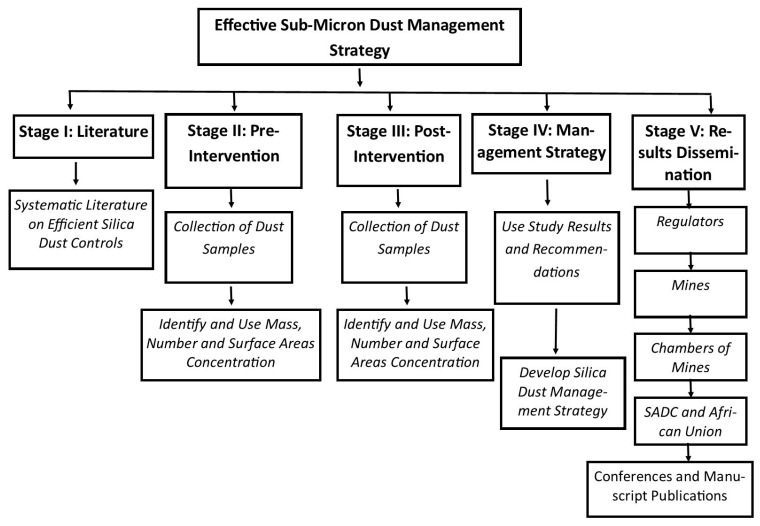
Research flowchart.

## Data Availability

The original contributions presented in this study are included in the article/supplementary material. Further inquiries can be directed to the corresponding author.

## Supplementary Materials

The following supporting information can be downloaded at https://www.mdpi.com/article/10.3390/mps9010012/s1. File S1: Research study informed consent information and form; File S2: the Workers Demographic Assumptions and Implications.
