# Supplementary material for: A Study Protocol for Developing a Pragmatic Aetiology-Based Silicosis Prevention and Elimination Approach in Southern Africa
Source: Methods Protoc. 2026 Jan 14;9(1):12. doi: 10.3390/mps9010012 (PMC12821596; doi:10.3390/mps9010012)
Supplement: Supplementary file 1 [file mps-09-00012-s001.zip › mps-3672762 File S1 Informed consent form.pdf]

File S1: Information Sheet and Informed Consent Form

23 May 2025

Good Day

My name is Norman Khoza **I WOULD LIKE TO INVITE YOU TO PARTICIPATE** in a research study on Systematic Management of Crystalline Silica Dust in Southern Africa: A Pragmatic etiologic-based silicosis prevention and elimination approach

Before you decide on whether to participate, I would like to explain to you why the research is being done and what it will involve for you. **I will go through the information letter with you and answer any questions you have.** This should take about 10 to 20 minutes. The study is part of a research project being completed as a requirement for a Doctoral Degree in Environmental Health through the University of Johannesburg.

THE PURPOSE OF THIS STUDY is to systematically investigate and develop the effectiveness of respirable dust and respirable crystalline silica dust exposure evaluation and control methodology for mining.

Below, I have compiled a set of questions and answers that I believe will assist you in understanding the relevant details of participation in this research study. Please read through these. If you have any further questions I will be happy to answer them for you.

1. **DO I HAVE TO TAKE PART?** No, you don't have to. It is up to you to decide to participate in the study. I will describe the study and go through this information sheet. If you agree to take part, I will then ask you to sign a consent form.

2. **WHAT EXACTLY WILL I BE EXPECTED TO DO IF I AGREE TO PARTICIPATE?** You will be requested to wear the personal sampling pump for the duration of your shift for a couple of days. You will also be request to complete an attitude, knowledge and practice questionnaire and any observation questions during data collection to supplement the data collected

3. **APPROXIMATELY HOW LONG WILL MY PARTICIPATION TAKE?** Your participation will take approximately This should take you about 30-45 minutes to complete the questionnaire and two days of sampling.

4. **WHAT WILL HAPPEN IF I WANT TO WITHDRAW FROM THE STUDY?** If you decide to participate, you are free to withdraw your consent at any time without giving a reason and without any consequences. If you wish to withdraw your consent, you should inform me as soon as possible.

5. **IF I CHOOSE TO PARTICIPATE, WILL THERE BE ANY EXPENSES FOR ME, OR PAYMENT DUE TO ME?** You will not be paid to participate in this study, and you will not bear any expenses

6. **IF I CHOOSE TO PARTICIPATE, WHAT ARE THE RISKS INVOLVED?** There are no risks foreseen by taking part in the study.

7. **IF I CHOOSE TO PARTICIPATE, WHAT ARE THE BENEFITS INVOLVED?** There are no immediate benefits, however, the study will develop strategies for the protection of employees against dust in small-scale mining industries and mining in general.

8. **WILL MY PARTICIPATION IN THIS STUDY BE KEPT CONFIDENTIAL?** All reasonable efforts will be made to keep your personal information confidential and respect your right to privacy. This includes replacing your identifying personal information with a number that only I and my research supervisor will know. You will not be identified in any research reports that are published. Under some circumstances, such as when required to do so by a court of law, I may have to disclose your personal information. In addition, it may happen that your information will need to be reviewed by another organisation for quality assurance purposes. I will tell you about this if it happens.

9. **WHAT WILL HAPPEN TO THE RESULTS OF THE RESEARCH STUDY?** The results will be written into a research report that will be assessed. In some cases, results may also be published in a scientific journal. In either case, you will not be identifiable in any documents, reports, or publications. You will be given access to the study results through various platforms within your mine.

10. **WHAT WILL YOUR RESPONSIBILITIES BE, AS THE RESEARCHER? To seek**

11. **WHO IS ORGANISING AND FUNDING THIS RESEARCH STUDY?** The study is being organised by me, under the guidance of my research supervisor at the Department of Environmental Health of the University of Johannesburg. The study forms part of a bigger project by the African Union Development Agency – New Partnership for Africa's Development (AUDA-NEPAD).

12. **WHO HAS REVIEWED AND APPROVED THIS STUDY?** Before this study was allowed to start, it was reviewed in order to protect your interests. This review was done first by the Department of Environmental Health, and then secondly by the Faculty of Health Sciences Research Ethics Committee at the University of Johannesburg. In both cases, the study was approved.

13. **WHAT IF THERE IS A PROBLEM?** If you have any concerns or complaints about this research study, its procedures or risks and benefits, you should ask me. You should contact me at any time if you feel you have any concerns about being a part of this study. My contact details are:

Norman Nkuzi Khoza
073 236 3424
normankhoza75@gmail.com

You may also contact my research supervisor:
Professor Daniel Masekameni: E-mail: daniel.masekameni@wits.ac.za
Professor Thokozani Mbonane: E-mail: tmbonane@uj.ac.za
Professor Phoka Rathebe: E-mail: prathebe@uj.ac.za

If you feel that any questions or complaints regarding your participation in this study have not been dealt with adequately, you may contact the Chairperson of the Faculty of Health Sciences Research Ethics Committee at the University of Johannesburg:

Prof. Christopher Stein
Tel: 011 559-6564
Email: cstein@uj.ac.za

FURTHER INFORMATION AND CONTACT DETAILS: Should you wish to have more specific information about this research project information, have any questions, concerns or complaints about this research study, its procedures, risks and benefits, you should communicate with me using any of the contact details given above.

Researcher:

Norman Khoza

DEPARTMENT OF ENVIRONMENTAL HEALTH
RESEARCH CONSENT FORM
REC 11.0

Systematic management of crystalline silica dust in Southern Africa: A pragmatic etiologic-based silicosis prevention and elimination approach

Please initial each box below:

☐ I confirm that I have read and understand the information letter dated 23 May 2025 for the above study. I have had the opportunity to consider the information, ask questions and have had these answered satisfactorily.

☐ I understand that my participation is voluntary and that I am free to withdraw from this study at any time without giving any reason and without any consequences to me.

☐ I agree to participate in the above research.

________________________ ____________________________________

Name of Participant | Signature of Participant | Date

________________________ ____________________________________

Name of Researcher | Signature of Researcher | Date
