# Supplementary material for: A Study Protocol for Developing a Pragmatic Aetiology-Based Silicosis Prevention and Elimination Approach in Southern Africa
Source: Methods Protoc. 2026 Jan 14;9(1):12. doi: 10.3390/mps9010012 (PMC12821596; doi:10.3390/mps9010012)
Supplement: Supplementary file 1 [file mps-09-00012-s001.zip › mps-3672762 File S2 Demographic assumptions and implications.pdf]

Table S2: Worker Demographic Assumptions and Implications

Demographic factor	Assumptions	Implications
Age	*Young workers are at an increased risk of injuries due to risk-taking behaviour and inexperience. Older workers are safer; however, they are more prone to diseases due to underlying health issues.*	*Training, mentorship, and supervision for young workers Detailed health risk assessment and awareness campaigns.*
Gender	*Males and females have unique occupational challenges, such as absent sanitary facilities, male-designed PPE and harassment.*	*Develop gender-specific or tailored OHS safety and health programs, and support infrastructure and PPE for women, as well as human rights awareness.*
Work experience (Knowledge and attitude)	*Inexperienced workers are more susceptible to injuries, while experienced workers may become complacent or develop a false sense of security.*	*Induction, safety toolboxes, refresher training and mentorship, including safety incentives.*
Work environment and culture (practices)	*Inadequate health and safety policies and procedures, Night shifts, long hours, a lack of supervision, or non-compliance with safety standards, as well as the improper use of personal protective equipment (PPE), can increase the risk of injury.*	*Institute H&S policies, awareness, supervision and monitoring, enforcement of safety rules, provision, maintenance and use of PPE. Visible felt leadership (VFL) model*
Socio-economic status	*Vulnerable workers, such as migrants, the disabled, and workers of low socioeconomic status, are prone to injuries, incidents and accidents.*	*Translated safety materials and outreach initiatives can aid in closing the communication gap.*
Physical attributes	*In some work contexts, employees with physical limitations, disabilities, or pre-existing medical issues may be at higher risk.*	*Ensure that the well-being of employees, accommodations, ergonomic evaluations, and customised safety strategies are essential.*
